# Feasibility and Preliminary Effects of Aquatic Exercise on Pulmonary Function and Dynamic Balance in Young Adult Smokers: A Pilot Randomized Controlled Trial

**DOI:** 10.3390/life16030379

**Published:** 2026-02-27

**Authors:** Ahmet Koyunlu, Zarife Pancar, Burak Karaca, Luca Russo

**Affiliations:** 1 Department of Coaching Education, Faculty of Sport Sciences, Kahramanmaraş Sütçü İmam University, Kahramanmaraş 46100, Turkey; koyunluahmet@gmail.com; 2 Department of Physical Education and Sports, Faculty of Sports Science, Gaziantep University, Gaziantep 27310, Turkey; 3 Department of Physical Education and Sports, Institute of Health Sciences, Gaziantep University, Gaziantep 27310, Turkey; burakkaracapt@gmail.com; 4 Department of Theoretical and Applied Sciences, eCampus University, Via Isimbardi 10, 22060 Novedrate, Italy; luca.russo2@uniecampus.it

**Keywords:** aquatic exercise, smoking, pulmonary function, dynamic balance, exercise intervention

## Abstract

Background: Smoking is a major public health concern worldwide and is associated with adverse effects on pulmonary function, postural control, and overall physical performance. Aquatic exercise has gained increasing attention as a safe and effective training modality due to its unique physical properties. However, evidence regarding the effects of aquatic exercise on pulmonary function and dynamic balance in young adult smokers remains limited. Objective: This study aimed to investigate the effects of an 8-week aquatic exercise training program on pulmonary function parameters and dynamic balance performance in young adult smokers. Methods: Twenty-two physically inactive male smokers were randomly assigned to an experimental group (*n* = 11) or a control group (*n* = 11). The experimental group participated in an aquatic exercise program three times per week for eight weeks, while the control group maintained their usual daily activities. Pulmonary function parameters, including FEV_1_, FVC, FEV_1_/FVC, PEF, PIF, MVV, VC, TV, and IVC, were assessed using spirometers. Dynamic balance performance was evaluated using a portable dynamic balance platform under single-leg (right and left) and double-leg conditions. Data were analyzed using a two-way repeated-measures ANOVA. Results: Statistically significant time × group interaction effects were observed for vital capacity (VC) (*p* = 0.033, η^2^*_p_* = 0.378) and tidal volume (TV) (*p* < 0.001, η^2^*_p_* = 0.734), suggesting potentially greater changes in the experimental group compared to the control group. Peak expiratory flow (PEF) demonstrated significant main effects of time (*p* = 0.047) and group (*p* = 0.031). Dynamic balance performance showed statistically significant time × group interaction effects across right-leg, left-leg, and bilateral conditions (*p* < 0.01), with large effect sizes (η^2^*_p_* = 0.762, 0.609, and 0.507, respectively). However, given the pilot nature and limited sample size of the study, these findings should be interpreted as preliminary. No significant changes were observed in FEV_1_, FVC, or FEV_1_/FVC ratio. Conclusions: This pilot randomized trial suggests that an 8-week aquatic exercise program is feasible and may produce preliminary improvements in selected pulmonary function parameters and dynamic balance in young adult smokers. Larger, adequately powered trials are required to confirm these findings.

## 1. Introduction

Cigarette smoking remains a major public health concern both nationally and globally, and the increasing prevalence of tobacco use has been accompanied by a marked rise in smoking-related health problems [1]. Smoking is widely recognized as one of the primary risk factors for the development of cardiovascular and respiratory diseases. Toxic components present in cigarette smoke induce structural and functional alterations in lung tissue, thereby adversely affecting pulmonary function [2]. These smoking-related physiological impairments not only compromise individuals’ health status but also negatively influence their participation in physical activity and overall quality of daily life [3,4,5]. Reductions in pulmonary capacity, early onset of fatigue, and decreased exercise tolerance make it increasingly difficult for individuals to maintain an active lifestyle, thereby promoting the adoption of sedentary behaviors in the long term. This condition compels individuals to cope with various health-related problems and further reinforces the negative cycle associated with smoking behavior [6]. Nicotine, one of the primary components of cigarette smoke, may induce transient neurological and vestibular symptoms, including dizziness, impaired balance, and nausea [7]. The stimulatory effects of nicotine on the central and peripheral nervous systems may lead to transient disruptions in postural control mechanisms, manifesting as dizziness and a sensation of imbalance. In addition, the adverse effects of nicotine on sensory systems and reflex responses may interfere with sensorimotor integration processes, potentially affecting postural regulation mechanisms. Evidence from the literature suggests that both acute nicotine exposure and chronic smoking can alter vestibular and neuromotor function. These alterations may contribute to disturbances in postural control over time [7,8,9].

Aquatic exercise has gained increasing attention in recent years due to its therapeutic and performance-enhancing properties [10]. The physical characteristics of water directly influence the physiological responses and motor activities elicited during exercise; in particular, buoyancy reduces the effects of gravity, thereby decreasing the mechanical load on the musculoskeletal system and making aquatic exercise a safe and effective rehabilitative modality [11]. In this context, aquatic plyometric training refers to explosive stretch–shortening cycle movements performed in a water environment, where buoyancy reduces impact forces and water resistance provides multidirectional external resistance. Owing to these properties, aquatic exercise has been reported to improve motor function and exert positive effects on balance performance [12,13]. Moreover, exercise performed in an aquatic environment may elicit greater increases in cardiac output and pulse pressure compared with land-based exercise [14]. Furthermore, when land-based exercise is perceived as monotonous or is not preferred for various reasons, aquatic exercise emerges as an effective and feasible alternative [15]. The physical properties of water, including buoyancy, hydrostatic pressure, viscosity, and turbulence, induce continuous micro-level body displacement during exercise. This environment provides ongoing stimulation to the balance control system, necessitating constant adjustments in postural stabilization. Through this mechanism, aquatic exercise has been shown to make a substantial contribution to the improvement of balance performance [16].

Aquatic exercise performed against water resistance provides multidirectional stimuli to the musculoskeletal system, thereby eliciting beneficial effects on various performance-related parameters [17]. In particular, aquatic plyometric exercises have been reported to be more effective than comparable land-based training modalities [18]. During such training, the increased effort required to overcome water resistance contributes to improvements in muscular strength and enhances the resilience of joint structures [19]. In addition, aquatic plyometric training programs have been shown to improve respiratory endurance and induce favorable adaptations in ventilatory capacity. Aquatic plyometric training refers to explosive, stretch–shortening cycle movements performed in a water environment, where buoyancy reduces ground impact forces and water resistance provides multidirectional external load. Unlike land-based plyometrics, the hydrostatic pressure exerted during water immersion increases respiratory muscle workload by applying external compression to the thoracic cavity. This increased inspiratory resistance may stimulate respiratory muscle activation and improve ventilatory efficiency. Additionally, the combined cardiovascular and neuromuscular demands of aquatic plyometric movements may enhance overall pulmonary function through improved oxygen utilization and respiratory muscle endurance [20]. Taken together, these findings suggest that aquatic exercise may positively influence not only musculoskeletal performance but also functional health indicators such as pulmonary function and balance control. In addition to its effects on motor control, the hydrostatic pressure exerted by water during immersion plays a crucial role in respiratory mechanics. Hydrostatic pressure increases external resistance against chest wall expansion, thereby elevating the workload of the inspiratory muscles. This increased respiratory muscle demand may stimulate adaptive responses, including enhanced inspiratory muscle strength and improved ventilatory efficiency. Furthermore, water immersion has been shown to influence thoracic blood volume and pulmonary circulation, potentially contributing to improved ventilation–perfusion matching [12,13,20]. These physiological mechanisms provide a plausible mechanistic basis for the potential beneficial effects of aquatic exercise on pulmonary function parameters and further strengthen the rationale underlying the present study’s hypothesis.

In this context, aquatic exercise-based training programs are expected to serve as effective preventive and rehabilitative interventions for individuals whose respiratory capacity and balance performance are adversely affected by smoking. Therefore, the aim of the present study was to investigate the effects of an 8-week aquatic exercise training program on pulmonary function and dynamic balance performance in young adult smokers. The present pilot randomized study aimed to explore the feasibility and preliminary effects of an 8-week aquatic exercise program on pulmonary function and dynamic balance in young adult smokers.

## 2. Materials and Methods

### 2.1. Study Design

This study was designed as a pilot randomized controlled trial to explore feasibility parameters (recruitment, adherence, safety, and protocol implementation) and to generate preliminary effect estimates for future adequately powered confirmatory trials. The training intervention of this study was conducted between March and May 2025. Prior to the commencement of the study, all participants were thoroughly informed about the purpose of the research and the procedures involved. The training methods to be applied, as well as the balance performance and pulmonary function tests, were introduced to the participants, and familiarization trials were performed before data collection to minimize potential measurement errors. The experimental group participated in an 8-week aquatic exercise training program, performed three times per week on nonconsecutive days. The control group did not receive any structured training intervention and was instructed to maintain their usual daily activities throughout the study period and refrain from initiating any new structured exercise program. Physical activity outside the intervention was not objectively monitored. The training program was initiated at a low intensity and progressively increased in accordance with the principle of gradual overload. Before each training session, a standardized warm-up protocol consisting of low-intensity running and stretching exercises was implemented to reduce the risk of injury.

To ensure the effectiveness of the aquatic training intervention, all exercises were conducted in a pool with a water depth not exceeding the participants’ body height. In addition, water-appropriate footwear with non-slip soles was used throughout the training sessions to minimize the risk of slipping. Prior to the intervention, baseline measurements of body weight, height, balance performance, and pulmonary function were obtained for all participants. Participants were randomly assigned to the experimental and control groups using a computer-based simple randomization method. This study was reported in accordance with the CONSORT (Consolidated Standards of Reporting Trials) guidelines for randomized controlled trials [21]. Ethical approval was obtained from the Kahramanmaraş Sütçü İmam University Medical Research Ethics Committee (approval date: 10 March 2025; decision number: 04), and all procedures were conducted in accordance with the principles of the Declaration of Helsinki. Written informed consent was obtained from all participants prior to their inclusion in the study.

### 2.2. Participants

A total of 22 young male individuals who were smokers and had no prior regular sports participation were included in the study. Physical activity status was determined through a structured verbal interview. Participants were classified as sedentary if they reported not engaging in any regular structured exercise or organized sports activity within the previous six months. Participants were randomly assigned to either the experimental group (*n* = 11; age [years]: 20.27 ± 2.10; height [cm]: 174.64 ± 7.00; body mass [kg]: 81.45 ± 11.70; smoking history [years]: 4.91 ± 1.51) or the control group (*n* = 11; age [years]: 20.73 ± 2.00; height [cm]: 175.45 ± 9.50; body mass [kg]: 81.18 ± 11.80; smoking history [years]: 4.73 ± 1.56). Sample size was calculated using G*Power software (version 3.1.9.4). Although an a priori power analysis was performed, the present study should be considered exploratory due to the limited sample size. The primary purpose was to estimate effect sizes and inform future sample size calculations rather than to provide definitive efficacy conclusions. An a priori power analysis was conducted using G*Power software (version 3.1.9.4). The calculation was based on the primary outcome variable: dynamic balance performance. A repeated-measures ANOVA (within–between interactions) was performed, with an alpha level of 0.05 and statistical power of 0.80. A large effect size (f = 0.40) was assumed, based on previous literature reporting substantial improvements in balance performance following aquatic-based interventions. The minimum required sample size was calculated as 20 participants. Therefore, 22 participants were included in the study. Given the relatively small sample size, the present study should be interpreted as a pilot randomized controlled trial. The inclusion criteria were as follows: (a) participation in both pre-test and post-test assessments; (b) attendance of at least 90% of the prescribed training sessions; and (c) absence of any injury, illness, or physical limitation that could hinder participation in the study. Smoking status was assessed through a structured verbal interview, and only individuals who reported active cigarette smoking for at least three years were included in the study. Participants were habitual daily smokers with a smoking history of 4.91 ± 1.51 years. Smoking status was assessed through a structured verbal interview. Based on self-reports, most participants indicated consuming approximately one pack of cigarettes per day; however, exact daily cigarette counts and pack-year calculations were not formally quantified.

### 2.3. Aquatic Training Protocol

Participants in the experimental group completed an 8-week aquatic plyometric training program performed three times per week (24 sessions total). The water temperature was maintained between 27 and 29 °C, and water depth corresponded to approximately chest level (xiphoid process level) for all participants. Each session consisted of approximately 45–50 min and was structured as follows:

Warm-up: 10 min of land-based mobility exercises followed by 5 min of low-intensity water-based warm-up activities.

Main set: Aquatic plyometric exercises performed in chest-deep water, including squat jumps, split squat jumps, tuck jumps, skater jumps, single-leg lateral hops, double-leg line jumps (forward/backward), and single-leg zig-zag hops.

Cool-down: 10 min of low-intensity movements and stretching exercises in water. Training volume progressed across the 8 weeks. During Weeks 1–4, participants performed 2–3 sets of 5–7 repetitions per exercise. During Weeks 5–8, volume increased to 2–3 sets of 10–12 repetitions per exercise. Rest intervals consisted of 30 s between sets and 60 s between exercises. Intensity was progressively increased through volume manipulation and exercise complexity. All sessions were supervised by a qualified instructor to ensure proper technique and adherence. Exercise intensity was monitored using the Borg 1–10 Rating of Perceived Exertion (RPE) scale. Participants were instructed to maintain an exertion level between 5 and 7 (moderate to vigorous intensity) during the main exercise phase, and RPE was recorded to ensure consistent training load control.

### 2.4. Measurements

Anthropometric Measurements: Participants’ body weight was measured using an electronic scale with a precision of 0.1 kg. Height was assessed using a stadiometer with a precision of 0.1 cm (SECA GmbH, Hamburg, Germany) [22,23]. All measurements were performed in accordance with standard measurement protocols, with participants wearing light clothing and no footwear.

Dynamic Balance Assessment: Dynamic balance performance was assessed using a portable dynamic balance device (Togu Challenge Disc 2.0, Prien am Chiemsee, Rosenheim, Germany). The platform allows free movement in all directions up to a maximum inclination of 12°, enabling the assessment of balance control under unstable surface conditions. The device records balance-related movements via three-dimensional sensors and transmits the data in real time to dedicated software through a Bluetooth connection. Balance performance was evaluated using a stability index ranging from 1 to 5, with lower scores indicating better balance performance. To eliminate the potential influence of footwear on balance outcomes, all measurements were performed barefoot. Participants were instructed to stand in the center of the disc with their arms free and maintain balance for 20 s while keeping the target displayed on the screen as stable and centered as possible. Each measurement was performed twice with a 3 min passive rest interval between trials, and the best score was used for statistical analysis [24,25,26].

Pulmonary Function Assessment: Pulmonary function was assessed using an open-circuit spirometry device equipped with disposable mouthpieces (MIR Spirodoc, Rome, Italy). All spirometric data were automatically recorded by the device during testing. Spirometry measurements were conducted in accordance with internationally accepted standard protocols. The following spirometric parameters were analyzed: forced vital capacity (FVC), forced expiratory volume in the first second (FEV_1_), FEV_1_/FVC ratio, peak expiratory flow (PEF), peak inspiratory flow (PIF), maximum voluntary ventilation (MVV), vital capacity (VC), tidal volume (TV), and inspiratory vital capacity (IVC) [27,28,29].

### 2.5. Statistical Analyses

Data were analyzed using SPSS for Windows (version 22.0; SPSS Inc., Chicago, IL, USA). Results are presented as the arithmetic mean ± standard deviation. The Shapiro–Wilk test was used to assess normality, and Levene’s test was used to evaluate homogeneity of variances. Skewness and kurtosis values were checked for each data set, and values within ±2 were considered to indicate normal distribution [30]. A two-way analysis of variance (2 × 2) with repeated measures was performed to analyze differences between groups over time. For cases where the assumption of sphericity was violated, the Greenhouse–Geisser correction was applied. The LSD test was used for post hoc analysis. Effect sizes (η^2^*_p_*) for ANOVA results were interpreted as minimal (η^2^*_p_* ≤ 0.02), moderate (0.02 < η^2^*_p_* ≤ 0.09), or strong (η^2^*_p_* > 0.09) [31]. Statistical significance was set at *p* < 0.05. All participants completed the study, and no missing data were recorded. Therefore, complete-case analysis was performed for all outcomes. No interim analyses were conducted.

## 3. Results

**Figure 1:**
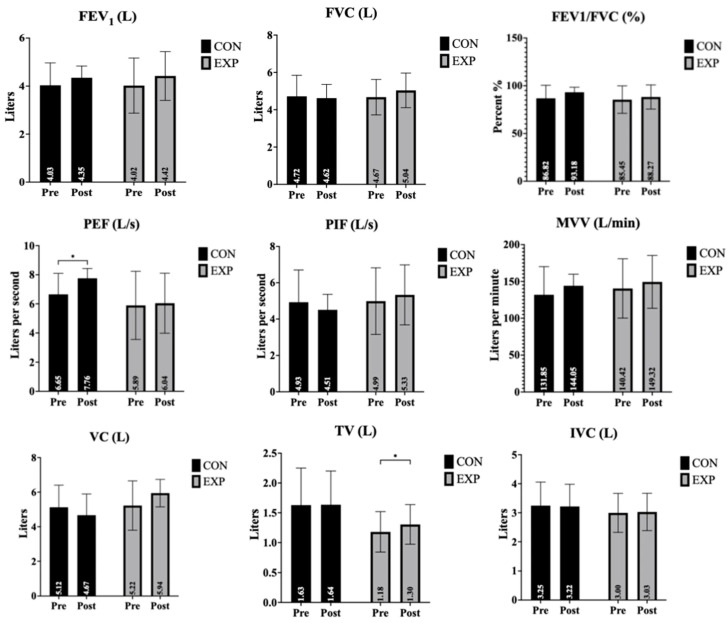
Changes in pulmonary function parameters before and after the intervention. Data are presented as mean ± standard deviation (SD). * *p* < 0.05 indicates a significant time × group interaction.

The effects of the intervention on pulmonary function parameters, including FEV_1_, FVC, FEV_1_/FVC ratio, PEF, PIF, MVV, and VC, for both the control and experimental groups are presented in Table 1 and Figure 1.

FEV_1_ (forced expiratory volume in 1 s): There was a near-significant main effect of time on FEV_1_ values, indicating a trend toward overall improvement across the sample. However, neither the main effect of group nor the time × group interaction reached statistical significance. These results suggest that changes in FEV_1_ were similar between the experimental and control groups.

FVC (forced vital capacity): FVC values showed no significant main effects for time, group, or interaction, indicating no substantial differences either over the course of the study or between the groups.

FEV_1_/FVC ratio: The FEV_1_/FVC ratio demonstrated a time effect that approached significance. No significant group effect or interaction effect was observed. Thus, while there was a general trend of improvement, no clear group-related difference was detected.

PEF (peak expiratory flow): PEF showed a significant main effect of time (and group). However, the interaction effect was not significant, indicating that changes over time were not statistically different between the groups. Post hoc analyses revealed a significant pre–post change across time (mean difference = −0.630, t = −2.267, *p* = 0.047). In addition, a significant between-group difference was observed, with higher PEF values in the experimental group compared to the control group (mean difference = 1.234, t = 2.510, *p* = 0.031).

PIF (peak inspiratory flow): For PIF, there were no significant effects for time, group, or interaction. Although group and interaction effect sizes were moderate, the observed differences did not reach statistical significance.

MVV (maximum voluntary ventilation): MVV did not demonstrate any significant main effect of time, group, or interaction, suggesting the intervention did not meaningfully affect this parameter.

VC (vital capacity): VC showed no significant time effect, but there were significant effects for group and for the time × group interaction. These findings indicate that the experimental group exhibited a greater increase in VC compared to the control group, with post hoc analysis confirming a significant pre- to post-test improvement within the experimental group (*p* < 0.05). Further analysis indicated that this interaction was primarily driven by a significant between-group difference observed at post-test (mean difference = −0.688, t = −2.419, *p* = 0.036).

TV (tidal volume): A significant main effect of time was found for TV, and the time × group interaction was highly significant. The group effect approached significance. Post hoc analysis revealed a significant increase in TV from pre- to post-intervention in the experimental group (*p* < 0.05), underscoring the intervention’s specific efficacy. Further examination of post hoc results showed that the observed effects were supported by a significant overall pre–post change in tidal volume (mean difference = −0.065, t = −2.977, *p* = 0.014), consistent with the strong interaction effect.

IVC (inspiratory vital capacity): There were no significant main effects for IVC in terms of time, group, or interaction. These results indicate that the intervention did not substantially affect inspiratory vital capacity.

**Figure 2:**
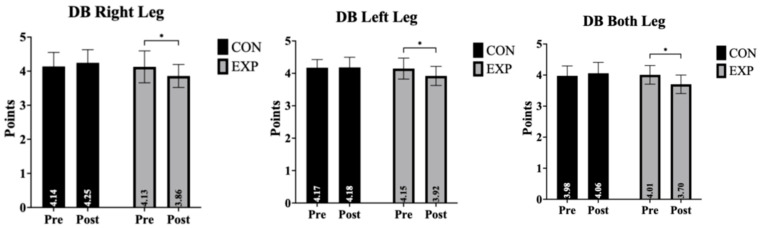
Pre- and post-intervention comparisons of dynamic balance performance for the right leg, left leg, and both legs in the control (CON) and experimental (EXP) groups. Data are presented as mean ± standard deviation (SD); * indicates a significant pre–post difference (*p* < 0.05).

Dynamic balance outcomes for the right leg, left leg, and both legs, including time, group, and time × group interaction effects, are presented in Table 2 and Figure 2. Dynamic balance outcomes are presented in Table 2 and Figure 2. Overall, significant group-by-time interaction effects were observed across right-leg, left-leg, and bilateral balance conditions. Post hoc analyses indicated that improvements from pre- to post-intervention occurred exclusively in the experimental group, whereas the control group showed no meaningful changes. These findings suggest that aquatic exercise may contribute to improvements in dynamic balance performance.

## 4. Discussion

The main findings of this study suggest that an 8-week aquatic exercise training program applied to young adult smokers may be associated with potentially beneficial effects, particularly on selected pulmonary function parameters (VC and TV) as well as dynamic balance performance. These results indicate that aquatic exercise may represent a feasible and promising intervention that could potentially help mitigate smoking-related impairments in respiratory function and postural control. Importantly, the present findings should be interpreted within the context of a pilot randomized design. The relatively small sample size limits statistical power and generalizability. Therefore, the observed improvements should be considered preliminary signals rather than confirmatory evidence of efficacy. The effect size estimates reported in this study may serve as a basis for planning future larger-scale randomized controlled trials.

In the present study, significant time × group interaction effects were observed in forced vital capacity (VC) and tidal volume (TV) in favor of the experimental group. These findings indicate that aquatic exercise may exert a facilitatory effect on ventilatory capacity. It is well established that exposure to cigarette smoke leads to reductions in lung volumes by decreasing alveolar elasticity and increasing airway resistance [32,33]. During exercise performed in an aquatic environment, hydrostatic pressure applied to the chest wall may increase the workload of the inspiratory muscles. This increased respiratory demand could contribute to ventilatory adaptations. However, respiratory muscle strength or endurance was not directly measured in the present study; therefore, these interpretations should be considered speculative and based on existing literature rather than direct empirical evidence [34,35,36,37,38]. This physiological mechanism may explain the improvements observed in VC and TV in the present study. In contrast, only limited or statistically non-significant changes were detected in FEV_1_, FVC, and the FEV_1_/FVC ratio. Although no significant changes were observed in FEV_1_, this may be related to the relatively short duration of the intervention. Structural airway remodeling and smoking-related airflow limitations typically require prolonged interventions or smoking cessation over several months or years to demonstrate measurable improvement. Therefore, the 8-week duration of the present study may have been insufficient to induce detectable changes in FEV_1_, particularly considering the chronic nature of smoking-related airflow alterations. This finding may be attributed to the relatively short duration of the intervention and the young age of the study population. Previous research has demonstrated that smoking-related obstructive changes become more pronounced following prolonged exposure and that improvements in parameters such as FEV_1_ often require longer-term interventions [39,40]. Therefore, the observation of only a trend toward improvement in FEV_1_ and related parameters in the present study suggests that aquatic exercise may preferentially influence lung volume- and breathing pattern-related parameters during the early stages of intervention. Previous evidence indicates that smoking is associated with reduced respiratory muscle strength and exercise performance in young adults, whereas higher levels of physical activity may attenuate these adverse effects [41]. In this context, aquatic exercise may represent an effective and low-impact strategy to counteract smoking-related functional impairments.

Another important finding of the present study is the pronounced and robust improvement in dynamic balance performance observed in the experimental group. The significant time × group interaction effects identified in right-leg, left-leg, and double-leg balance assessments indicate that aquatic exercise provides a strong stimulus to postural control mechanisms. Previous studies have reported that smoking adversely affects the vestibular system, proprioceptive feedback, and central nervous system integration, thereby impairing balance performance [8,42,43]. In particular, the effects of nicotine on vestibular nuclei and reflex pathways may contribute to deficits in postural stability [43].

Exercise performed in an aquatic environment exposes the body to continuous micro-level instability arising from the buoyancy, viscosity, and turbulence properties of water, requiring ongoing neuromuscular and sensory adjustments to maintain postural control. This environment may promote increased engagement of proprioceptive, vestibular, and central sensorimotor integration processes, potentially contributing to improvements in balance regulation [44,45,46,47]. In addition, the hydrostatic pressure exerted by water may increase inspiratory workload during exercise. However, respiratory muscle strength and endurance were not directly assessed in the present study; therefore, any interpretation regarding respiratory muscle adaptations should be considered speculative and based on existing literature rather than direct empirical evidence [34,48]. Although large effect sizes were observed for dynamic balance parameters, these findings should be interpreted cautiously, as clinical thresholds for meaningful change were not established in the present sample. While previous research has reported beneficial effects of aquatic exercise on balance and motor control in various populations [49,50,51], evidence in young adult smokers remains limited. Thus, the present findings provide preliminary evidence that aquatic exercise may support functional adaptations in this population. However, these results do not constitute clinical therapeutic recommendations and require further validation in larger and more diverse samples. In this regard, the present study makes a novel contribution to the literature by demonstrating that smoking-related functional impairments may be partially mitigated through aquatic exercise interventions. In particular, for young individuals who smoke but have not yet developed advanced pulmonary pathology, early implementation of aquatic exercise programs may be considered a preventive and rehabilitative strategy.

The present study has several limitations that should be carefully considered when interpreting the findings. First, the relatively small sample size limits statistical power and generalizability; therefore, the results should be considered preliminary. Second, the study included only a non-exercising control group and did not incorporate a land-based exercise comparison group. Consequently, it is not possible to determine whether the observed effects are specific to the aquatic environment or reflect general exercise-induced adaptations. Third, smoking exposure was assessed through self-reported duration and approximate daily consumption, without precise pack-year calculation or biochemical verification (e.g., exhaled CO or serum cotinine), which may have introduced reporting bias. In addition, respiratory muscle strength and endurance were not directly measured; therefore, mechanistic interpretations regarding respiratory muscle adaptations remain speculative. Fourth, dynamic balance was assessed using a single validated device without additional objective verification, which may limit measurement robustness. Moreover, multiple pulmonary parameters were analyzed, increasing the potential risk of Type I error despite statistical control procedures. Only young adult male participants were included, which limits generalizability to females and other age groups. Furthermore, daily physical activity levels of the control group were not objectively monitored, which may represent a potential confounding factor. Finally, the relatively short duration of the intervention may have limited detectable changes in certain pulmonary parameters. Therefore, the findings of the present study should be interpreted as exploratory and hypothesis-generating rather than definitive evidence of clinical efficacy. Future research should incorporate larger, more diverse samples, longer intervention periods, active comparison groups, and direct mechanistic assessments to strengthen methodological rigor and external validity.

## 5. Conclusions

The findings of this pilot study suggest that an 8-week aquatic exercise training program in young adult smokers may be associated with potentially beneficial changes in selected pulmonary function parameters and dynamic balance performance. In particular, the observed increases in vital capacity (VC) and tidal volume (TV) in the experimental group may indicate a possible improvement in ventilatory capacity. In contrast, the limited changes observed in FEV_1_, FVC, and the FEV_1_/FVC ratio may be related to the relatively short intervention duration and the young age of the participants. Regarding dynamic balance, statistically significant interaction effects were observed; however, these findings should be interpreted as preliminary, given the pilot nature of the study. The aquatic environment may provide proprioceptive and vestibular stimuli that could potentially contribute to postural control adaptations, although mechanistic interpretations remain speculative. Overall, aquatic exercise appears to be a feasible and well-tolerated intervention in this population. However, the present findings should not be interpreted as clinical recommendations or definitive evidence of efficacy. Instead, they should be considered hypothesis-generating observations that may inform the design of future adequately powered randomized controlled trials. Future research incorporating larger sample sizes, longer intervention periods, and active comparison groups is required to confirm and extend these preliminary findings.

## Figures and Tables

**Table 1:** Descriptive statistics and repeated-measures ANOVA results for pulmonary function parameters in control and experimental groups.

	Control		Experimental		*p* (F)		
	Pre	Post	Pre	Post	Time	Trial	Int. Eff.
FEV_1_	4.03 ± 0.93	4.35 ± 0.49	4.02 ± 1.15	4.42 ± 1.02	0.082	0.891	0.784
95% CI:	3.40–4.66	4.02–4.68	3.25–4.79	3.74–5.10	(3.740)	(0.020)	(0.079)
**η^2^** ***_p_*** **:**					0.272	0.002	0.008
FVC	4.72 ± 1.13	4.62 ± 0.74	4.67 ± 0.95	5.03 ± 0.93	0.627	0.343	0.351
95% CI:	3.96–5.47	4.13–5.11	4.04–5.31	4.41–5.66	(0.251)	(0.991)	(0.955)
**η^2^** ***_p_*** **:**					0.024	0.090	0.087
FEV_1_/FVC	86.82 ± 13.79	93.18 ± 5.27	85.45 ± 14.36	88.27 ± 12.64	0.084	0.251	0.351
95% CI:	77.55–96.08	89.64–96.72	75.81–95.10	79.78–96.77	(3.675)	(1.483)	(0.957)
**η^2^** ***_p_*** **:**					0.269	0.129	0.087
PEF	6.65 ± 1.44	7.75 ± 0.68	5.89 ± 2.34	6.04 ± 2.06	0.047	0.031	0.192
95% CI:	5.68–7.62	7.30–8.21	4.32–7.47	4.66–7.43	(5.137)	(6.300)	(1.955)
**η^2^** ***_p_*** **:**					0.339	0.387	0.164
PIF	4.92 ± 1.78	4.51 ± 0.85	4.99 ± 1.84	5.33 ± 1.65	0.911	0.183	0.134
95% CI:	3.73–6.12	3.93–5.08	3.75–6.22	4.22–6.44	(0.013)	(2.047)	(2.661)
**η^2^** ***_p_*** **:**					0.001	0.170	0.210
MVV	131.85 ± 38.26	144.05 ± 15.71	140.42 ± 40.32	149.32 ± 35.89	0.186	0.230	0.769
95% CI:	106.15–157.56	133.50 ± 154.61	113.33–167.51	125.21–173.43	(2.019)	(1.635)	(0.091)
**η^2^** ***_p_*** **:**					0.168	0.141	0.009
VC	5.12 ± 1.28	4.67 ± 1.23	5.22 ± 1.43	5.94 ± 0.79	0.764	0.036	0.033
95% CI:	4.26–5.98	3.84–5.49	4.26–6.18	5.41–6.48	(0.095)	(5.854)	(6.071)
**η^2^** ***_p_*** **:**					0.009	0.369	0.378
TV	1.63 ± 0.62	1.63 ± 0.56	1.18 ± 0.34	1.30 ± 0.33 *	0.014	0.075	<0.001
95% CI:	1.21–2.05	1.26–2.01	0.95–1.41	1.08–1.53	(8.863)	(3.940)	(27.651)
**η^2^** ***_p_*** **:**					0.470	0.283	0.734
IVC	3.25 ± 0.81	3.22 ± 0.76	3.00 ± 0.67	3.03 ± 0.64	0.927	0.575	0.354
95% CI:	2.70–3.79	2.70–3.73	2.55–3.45	2.60–3.46	(0.009)	(0.336)	(0.943)
**η^2^** ***_p_*** **:**					0.001	0.032	0.086

Abbreviations: FEV_1_ = forced expiratory volume in one second; FVC = forced vital capacity; FEV_1_/FVC = ratio of forced expiratory volume in one second to forced vital capacity; PEF = peak expiratory flow; PIF = peak inspiratory flow; MVV = maximum voluntary ventilation; VC = vital capacity; TV = tidal volume; IVC = inspiratory vital capacity; CI = confidence interval; Int. Eff. = interaction effect; η^2^*_p_* = partial eta squared. * Significant difference between pre- and post-tests.

**Table 2:** Descriptive statistics and repeated-measures ANOVA results for dynamic balance scores in control and experimental groups.

	Control		Experimental		*p* (F)		
	Pre	Post	Pre	Post	Time	Trial	Int. Eff.
Right Leg	4.14 ± 0.41	4.24 ± 0.39	4.13 ± 0.47	3.86 ± 0.34 *	0.115	0.023	<0.001
95% CI:	3.86–4.42	3.99–4.50	3.81–4.44	3.63–4.09	(2.978)	(7.180)	(32.063)
**n^2^** ***p*** **:**					0.229	0.418	0.762
Left Leg	4.17 ± 0.25	4.18 ± 0.31	4.15 ± 0.32	3.92 ± 0.29 *	0.035	0.147	0.003
95% CI:	4.00–4.34	3.97–4.39	3.93–4.36	3.72–4.12	(5.955)	(2.472)	(15.553)
**n^2^** ***p*** **:**					0.373	0.198	0.609
Both Leg	3.98 ± 0.32	4.06 ± 0.35	4.01 ± 0.30	3.70 ± 0.30 *	0.099	0.042	0.009
95% CI:	3.76–4.19	3.82–4.29	3.81–4.21	3.50–3.90	(3.311)	(5.415)	(10.265)
**n^2^** ***p*** **:**					0.249	0.351	0.507

Note. Dynamic balance scores represent the stability index (range: 1–5), expressed as a unitless index. Lower scores indicate better balance performance. * Significant difference between pre- and post-tests. Int. Eff: interaction effect. CI: confidence interval.

## Data Availability

The data obtained in this study are presented in tables within the article. The raw data are available from the corresponding author upon reasonable request. The data are not publicly available due to ethical and privacy restrictions.
